# Are Hyperglycemia-Induced Changes in the Retina Associated with Diabetes-Correlated Changes in the Brain? A Review from Zebrafish and Rodent Type 2 Diabetes Models

**DOI:** 10.3390/biology13070477

**Published:** 2024-06-27

**Authors:** Kaylee Augustine-Wofford, Victoria P. Connaughton, Elizabeth McCarthy

**Affiliations:** 1 Department of Biology, American University, Washington, DC 20016, USA; kayleeaugustinewofford@gmail.com (K.A.-W.); em3588a@american.edu (E.M.); 2 Center for Neuroscience and Behavior, American University, Washington, DC 20016, USA

**Keywords:** cognition, retinopathy, *Danio rerio*, *db*/*db* mouse, GK rats, ZDF rats

## Abstract

**Simple Summary:**

Diabetes is a worldwide epidemic that affects the retina, kidneys, and peripheral nerves. More recent findings indicate that brain changes also occur and are associated with dementia and/or cognitive deficits. As both the retina and the brain have blood–tissue barriers that are sensitive to high blood sugar levels, it has been proposed that complications in the retina, an extension of the brain, could be used to predict subsequent brain pathology. In this review, we summarize published data about the disease-associated changes in the retina and brain reported in animal models (rodents and zebrafish) to assess specific cellular and/or behavioral mechanisms associated with vision and cognitive function loss in diabetes.

**Abstract:**

Diabetes is prevalent worldwide, with >90% of the cases identified as Type 2 diabetes. High blood sugar (hyperglycemia) is the hallmark symptom of diabetes, with prolonged and uncontrolled levels contributing to subsequent complications. Animal models have been used to study these complications, which include retinopathy, nephropathy, and peripheral neuropathy. More recent studies have focused on cognitive behaviors due to the increased risk of dementia/cognitive deficits that are reported to occur in older Type 2 diabetic patients. In this review, we collate the data reported from specific animal models (i.e., mouse, rat, zebrafish) that have been examined for changes in both retina/vision (retinopathy) and brain/cognition, including *db*/*db* mice, Goto-Kakizaki rats, Zucker Diabetic Fatty rats, high-fat diet-fed rodents and zebrafish, and hyperglycemic zebrafish induced by glucose immersion. These models were selected because rodents are widely recognized as established models for studying diabetic complications, while zebrafish represent a newer model in this field. Our goal is to (1) summarize the published findings relevant to these models, (2) identify similarities in cellular mechanisms underlying the disease progression that occur in both tissues, and (3) address the hypothesis that hyperglycemic-induced changes in retina precede or predict later complications in brain.

## 1. Introduction

Diabetes mellitus (DM) is a metabolic disorder characterized by elevated blood glucose levels (hyperglycemia) resulting from either insufficient insulin production (Type 1) or impaired insulin sensitivity (Type 2). DM affects >500 million people, with Type 2 accounting for more than 90% of individuals diagnosed every year [1]. Hyperglycemia associated with diabetes leads to visual complications and an increased risk of cognitive deficits/dementia [2,3,4,5,6,7]. The visual complications are clinically diagnosed by alterations in retinal vasculature [8,9,10,11,12], while the specific mechanisms underlying cognitive impairments remain less elucidated. However, parallels in pathological and cellular changes between the brains of diabetics and the brains of patients with Alzheimer’s disease/dementia suggest common mechanisms [7,13].

Diabetic retinopathy (DR) is a prevalent microvascular complication found in DM patients and a universal contributor to vision loss [14,15]. Prolonged/uncontrolled hyperglycemia, the most consistent risk factor for DR [16], triggers a pro-inflammatory response [17,18,19] leading to deterioration of the blood-retinal barrier (BRB) [20], a reduction in the number of tight junction proteins [21,22,23,24,25,26], a loss of pericytes [27,28,29,30,31], and an increase in vascular permeability [32]. DR is also neurodegenerative, resulting in functional deficits in electroretinogram (ERG) recordings [10,33,34] and the gradual loss of retinal neurons [35]. While changes in retinal vasculature are the clinical hallmark of DR [5], work in animal models [8,10,11,12,36] and humans [37,38] suggests that deficits in retinal function occur prior to noticeable vascular changes. 

Individuals with Type 2 Diabetes (T2DM) are at an increased risk of developing dementia, including Alzheimer’s disease [4,7,39] with cognitive impairment that is consistently noted in Type 2 diabetics [39,40]. While variations in the degree of impairment exist [40], deficits generally arise from cellular changes that are initially triggered by dysregulation of blood glucose levels [39,41,42,43]. These changes exacerbate normal age-related cognitive decline [7] and are associated with brain atrophy [44]. Thus, glucose-induced changes to the blood–brain barrier (BBB) are suggested to specifically drive changes in brain tissue. This statement is supported by the common structural composition of the BBB and the BRB and their heightened sensitivity to hyperglycemic insult [3,45]. Further, evidence from animal models suggests that increased BRB permeability may serve as an indicator of BBB damage [46,47] and/or memory impairment [46,48,49]. Examination of brain tissue from both diabetic patients and individuals with Alzheimer’s disease reveals an increase in inflammation, apoptosis, mitochondrial dysfunction, and protein aggregation [7], which are also observed in diabetic retinas, reinforcing this hypothesis. Postmortem examination of brain tissue also identifies thickened basement membranes in cerebral blood vessels [39] and an increase in BBB permeability in individuals with late-life cognitive decline [50,51]. 

DR has been studied primarily in rodents, with DM occurring either spontaneously via genetic elements or artificially via diet or streptozotocin injection. For both, DR is characterized by changes in retinal physiology [8,52,53,54], thinning of the retina [10], an increase in apoptosis [55,56,57], and damage to retinal vessels [58,59]. The use of zebrafish to study DM and its complications began in 2007, when alternate immersion in a glucose solution was found to increase blood glucose levels for 1 month [60]. Since then, the use of zebrafish to assess DM complications has increased as noted in several recent reviews [61,62,63,64,65,66,67,68,69]. This surge is primarily attributed to three characteristics of zebrafish: (1) their prominent use in molecular studies, including the generation of mutant and transgenic lines, (2) their ability to produce large numbers of offspring at a given time which develop externally, and (3) their remarkable parallels with humans. Zebrafish possess an insulin-secreting pancreas that exhibits morphological similarities to that of humans, including exocrine and endocrine compartments with comparable cellular composition [70]. Zebrafish eyes are structurally similar to human eyes, with key ocular components such as the cornea, lens, vitreous, retina, pigment epithelium, choroid, and sclera [20,71], as well as a predominance of cone photoreceptors reminiscent of those found in the central human retina [71]. Zebrafish also show notable genetic correspondence with humans, with an overlap of nearly 70% in genes associated with diabetes, some of which have been identified and cloned in zebrafish [72]. Further, the zebrafish BRB [73] and BBB [74] exhibit characteristics similar to those in higher vertebrates. Retinal vessels in zebrafish are in direct contact with Muller cell endfeet [73] and vascular endothelial cells are connected via junctional complexes and surrounded by pericytes [73]. Hyperglycemia, induced by either bath glucose exposure [60,75,76,77,78] or streptozotocin injections [79,80], thins the inner retina [60,80], thickens retinal vessel basement membranes, reduces tight junction proteins, increases the expression of proinflammatory cytokines [3,22], and alters retinal physiology [81]. Additionally, cone morphology changes [75] and glycated proteins are formed [77], features consistent with both rodent models of DR and human observations. 

Animal models of DM, including hyperglycemic zebrafish [76,82,83], also display learning and memory deficits [42]. Diets consisting of foods high in saturated fats and sugar that contribute to obesity and T2DM in humans disrupt BBB function and upregulate inflammatory markers in rodent brains, particularly in regions like the hippocampus, that underlie important higher-order cognitive processes [84,85,86]. These pathophysiological signs are associated with impairments in spatial learning and memory, discrimination reversal, and other behavioral paradigms [85,87,88].

Here, we review the current literature from zebrafish and rodent models targeting complications related to visual and cognitive deficits in T2DM. Our goal is to identify hyperglycemia-induced changes in the retina and the brain, based on cellular and/or behavioral data, to determine the current consensus with regard to the timing, impact, and common mechanisms that are present. We focus on T2DM because of its prevalence in the population and the increased occurrence of cognitive impairments in these individuals. We discuss information from four rodent models, with three genetic models that spontaneously develop T2DM (*db*/*db* mice, Goto-Kakizaki rats, and Zucker Diabetic Fatty rats) and one induced model (high fat diet ± streptozotocin), and with two T2DM induced zebrafish models (glucose immersion and diet induced obesity). We selected these models because they have been used to assess hyperglycemia-induced complications in both retina/vision and brain/cognition, allowing comparisons to be made between these tissues. 

## 2. Hyperglycemia-Induced Changes in Retina

### 2.1. Rodent Models

#### 2.1.1. Genetic/Spontaneously Occurring T2DM

**Figure 1:**
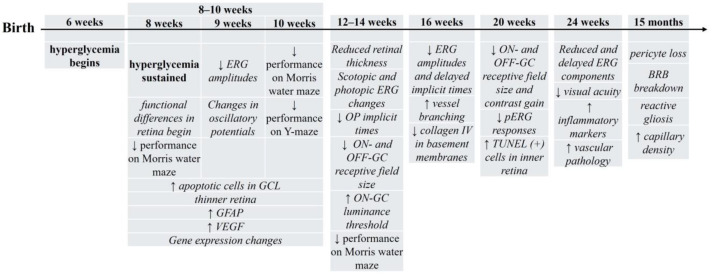
Timeline of hyperglycemia-induced changes in *db*/*db* mice, a genetic rodent model of T2DM. For the figure above, the timeline begins with birth (at the left). Age after birth (above the line in weeks or months) indicates when retinal and/or brain changes were assessed. Below the line/arrow are specific outcomes reported at each age. *db*/*db* mice become hyperglycemic at 6 weeks of age and this model has been assessed to 15 months of age. Outcomes in italics reflect retinal/vision changes; non-italic text reflects the changes in brain/cognition. ↑ = increase; ↓ = decrease. See text for details.

The prominent mouse model used to study T2DM complications is the leptin-receptor deficit *db*/*db* mouse [89]. These mice show progressive weight gain/obesity, hyperglycemia [52,55,90,91,92] beginning at 6 weeks of age [89], and an increase in HbA1c levels [10]. At 8 weeks of age, hyperglycemia is sustained [93] and functional differences begin to be evident [93,94,95]. Noticeable reductions in ERG b-wave amplitudes and changes to oscillatory potentials (OPs) occur at 9 weeks [10,96]. Cellular differences are also noticeable at 8–10 weeks of age including thinner retinal layers [38], an increase in the number of apoptotic cells in the ganglion cell layer [55], an increase in GFAP [55,97], upregulation of VEGF levels [97], and changes in gene expression [98] (Figure 1).

By 12–14 weeks of age (~3 months), *db*/*db* retinal thickness is still reduced [10], scotopic ERG a-waves increase slightly [99], and implicit times for OP1 and OP2 are delayed [91]. Significant reductions in cone (photopic) ERG amplitudes also occur [93]. 

In addition, ON- and OFF-ganglion cell (GC) receptive fields are smaller, ON-GC luminance thresholds increase [94], and pattern ERGs, which assess GC function, are slightly reduced [100]. Interestingly, despite these functional differences, there is only a slight deficit in visual acuity at this age [12].

With age and continued hyperglycemia, *db*/*db* retinas progressively deteriorate so that the decrease in ERG a-wave and/or b-wave amplitudes are evident by 16 weeks (~4 months), and often accompanied by delayed implicit times [52,55,93,99]. Retinal vasculature shows increased branching [59] and loss of collagen IV in basement membranes [58]. At 20 weeks, the receptive field sizes of both ON- and OFF-GC are decrease by ~60%, contrast gain is significantly reduced [94], and pattern ERGs show a reduced GC function [100]. There is also an increase in TUNEL(+) cells in the ganglion cell and the inner nuclear retinal layers [100]. Reduced and delayed ERG components are still evident at 6 months (~24 weeks) of age [10,55,93,100,101] along with a decrease in visual acuity [12], an increase inflammatory markers [100], and vascular changes [101]. Thinner retinas, lower numbers of GC, and an increase in apoptosis continues and worsens at 32 weeks of age [57]. By 15 months, the oldest age reported, vascular changes are significant and include pericyte loss, breakdown of the BRB, apoptosis, reactive gliosis, and an increase in capillary density [102].

**Figure 2:**
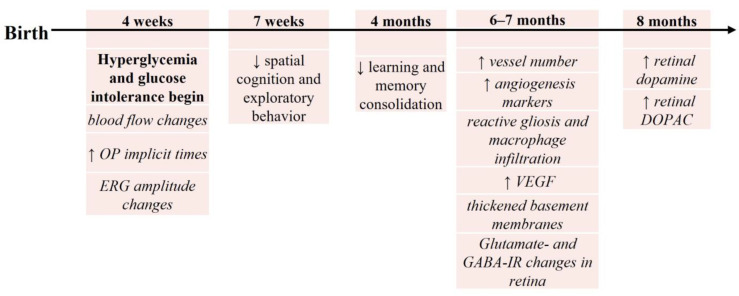
Timeline of hyperglycemia-induced changes in GK rats, a genetic rodent model of T2DM. For the figure above, the timeline begins with birth (at the left). Age after birth (above the line in weeks or months) indicates when retinal and/or brain changes were assessed. Below the line/arrow are specific outcomes reported at each age. Goto-Kakizaki (GK) rats are hyperglycemic at 4 weeks of age, with studies reported through 8 months of age. Outcomes in italics reflect retinal/vision changes; non-italic text reflects the changes in brain/cognition ↑ = increase; ↓ = decrease. See text for details.

Goto-Kakizaki (GK) rats also spontaneously develop T2DM [103]; however, these animals are non-obese with stable, moderate hyperglycemia [104,105,106]. At 1 month (4 weeks) of age, when hyperglycemia onset and glucose intolerance occur, retina perfusion shows a prolonged mean circulation time and a decrease in segmental blood flow [106], though acellular capillaries are not evident [95]. However, there are functional delays in flicker response and OP implicit times at this age [95,107]. Some reports also indicate deficits in ERG a-wave, b-wave, and OP amplitudes [34] at 1 month; while others report an increase in ERG amplitudes [95,107]. Between 6 and 7 months, vessel number and expression of angiogenic markers increase, suggesting neovascularization [108]. Additionally, there are signs of reactive gliosis, macrophage infiltration, upregulation of VEGF, and thickening of basement membranes [109] without a change in retinal thickness [106,110]. Shifts in glutamate- and GABA-immunoreactivity patterns occur at 28 weeks (~7 months) of age [110] and an increase in overall dopamine content [95] is apparent at 8 months (Figure 2), both potentially contributing to the observed physiological changes.

**Figure 3:**
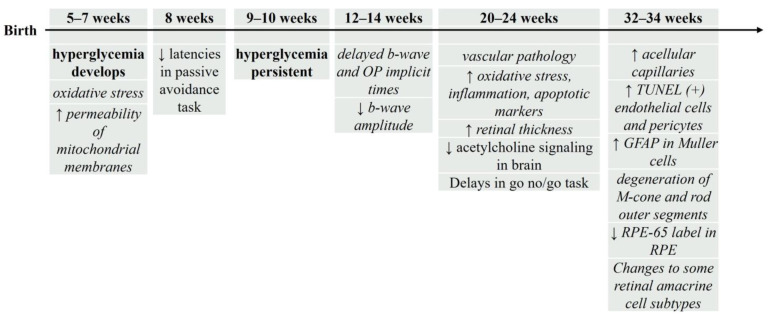
Timeline of hyperglycemia-induced changes in ZDF rats, a genetic rodent model of T2DM. For the figure above, the timeline begins with birth (at the left). Age after birth (above the line in weeks) indicates when retinal and/or brain changes were assessed. Below the line/arrow are specific outcomes reported at each age. Zucker Diabetic Fatty (ZDF) rats are consistently hyperglycemic at 9–10 weeks of age; this model has been studied until 32–34 weeks of age. Outcomes in italics reflect retinal/vision changes; non-italic text reflects the changes in brain/cognition. ↑ = increase; ↓ = decrease. See text for details.

Zucker Diabetic Fatty (ZDF) rats have a mutation in the leptin (*fa*/*fa*) gene causing them to gain weight and develop insulin resistance [36], making them a model of non-insulin dependent diabetes with obesity [111,112]. ZDF rats begin to develop hyperglycemia at 5–7 weeks of age (Figure 3), with significant and sustained hyperglycemia after 9–10 weeks [19,33,113,114]. A recent study [19] examined progressive changes in ZDF retinas by comparing 6-week-old (prediabetic), 12-week-old (moderate lipidemia, severe hyperglycemia), and 20–40-week-old (severe lipidemia and hyperglycemia) tissues. Their findings indicate a progression of complications beginning with early oxidative stress and changes to the permeability of mitochondrial membranes (6 weeks) followed by reduced ERG b-wave amplitudes (12 weeks) and subsequent vascular differences (20 weeks). Importantly, a difference from controls, once identified, persisted with age.

In agreement with the above study, others report changes to ERG recordings at 12–14 weeks of age [33] in ZDF retinas. These ERG differences precede morphological differences, such as increases in oxidative stress, inflammation, apoptotic markers [115,116], and retinal thickness [113], which appear at 20–24 weeks [113]. By 32–34 weeks (~8 months) of age, there is an increase in acellular capillaries and TUNEL(+) endothelial cells and pericytes [117,118], indicating vascular pathology. Using cell-specific antibodies to assess individual retinal cell types reveals morphological changes at 32 weeks, including an increase in GFAP expression in Muller glia, the degeneration of M-cone and rod outer segments, a reduction in RPE-65 labeling in the pigment epithelium, and changes to specific amacrine cell subtypes [114]. 

Overall, the above rodent models reveal striking similarities in T2DM-related retinopathy. *db*/*db* mice and ZDF rats are both obese and hyperglycemic at around the same age. This similarity seems logical given that the mutations underlying both these strains involve leptin. GK rats, on the other hand, are not obese, and develop hyperglycemia at a much earlier age. GK and ZDF rat models both display a more delayed progression of retinopathy than *db*/*db* mice, with sequelae taking months vs. weeks to appear. Despite these differences, retinopathy in all three models follows the same general timeline, with functional deficits appearing prior to vascular changes (as in [8,10,11,12,36,119]).

#### 2.1.2. Induced/Non-Genetic DM

Obesity is often comorbid with T2DM. In animal models, obesity is typically induced through consumption of a high fat diet (HFD), where 25–60% of the calories consumed are from fat [17]. Practical and relevant to this review is the use of a HFD (1) with both rats and mice and (2) in conjunction with low dose streptozotocin (STZ) injection. Importantly, HFD alone exacerbates complications of DM, particularly in the retina, and worsens risk factors for DR [17]. Consumption of a HFD for at least 2 months by C57Bl/6 male mice, Sprague-Dawley rats, or Wistar male rats increases body weight, fat deposits, triglyceride levels, total cholesterol, blood sugar, and insulin resistance [17,18,120,121,122,123,124]. Compared to genetic models, HFD-induced retinal changes take longer to appear [8]. By increasing free fatty acids [120,122] and altering lipid metabolism in retina, HFD triggers inflammation [17], similar to the early hyperglycemia-induced changes observed in ZDF rats. 

**Figure 4:**
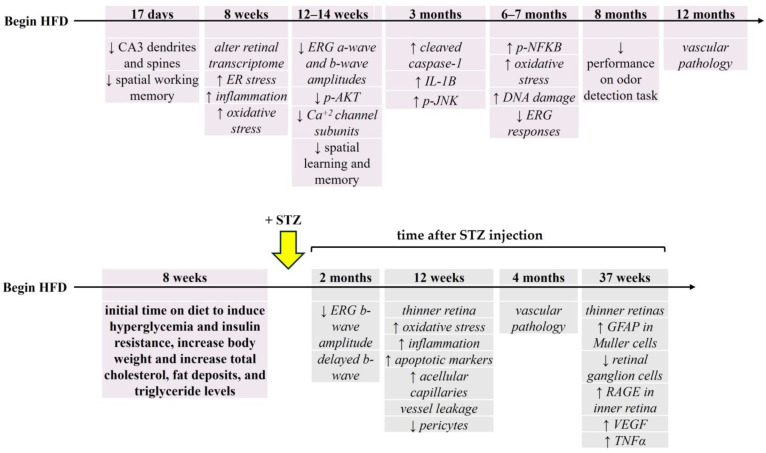
Hyperglycemia-induced changes in non-genetic/induced rodent models of T2DM. Rodents are fed a high fat diet (HFD) to induce hyperlipidemia and hyperglycemia. Subsequent examination of retina/vision and brain/cognition differences are assessed based on total duration on the diet. For each panel above, the timeline begins with onset of the HFD. Time on the diet (above the line in weeks or months) is used to determine diet-induced effects. Below the line/arrow are outcomes reported at each time point. In response to HFD alone (top), differences in the brain are observed as early as 17 days on the diet. Retinal differences, in contrast, are observed after 8 weeks on the diet. Alternatively (bottom panel), low dose streptozotocin (STZ, yellow arrow) is administered after time on the HFD. In this paradigm, changes to the retina are assessed at given time points after STZ injection. For both panels, outcomes in italics reflect retinal/vision changes; non-italic text reflects the changes in brain/cognition. ↑ = increase; ↓ = decrease. See text for details.

HFD-fed mice retinas show an increase in inflammation (p-NF-κB levels), oxidative stress, and DNA damage after 6 months on the diet [125]. ERG deficits are occur at 6 months [8], though they are initially apparent after only 12 weeks on the HFD [54] (Figure 4). HFD-fed rodents also display reductions in p-AKT levels and calcium channel subunits after 12 weeks [54], the latter suggesting a potential mechanism for the smaller ERG a-wave and b-wave responses at this time. Apoptotic markers (i.e., cleaved caspase-1, IL-B, p-JNK) increase after 3 months on the HFD [119], when a thinner retina is observed [122]. A shorter diet duration (8 weeks) affects the retinal transcriptome [126] and induces ER stress resulting in Muller glia dysregulation and inflammation [121,123]. Consistent with genetic T2DM models, vascular deficits are delayed in appearance and not evident until after 12 months on the diet in HFD-fed mouse retinas [8,119]. 

Consumption of a HFD leads to hyperinsulinemia but not necessarily insulin resistance [124], the latter being a characteristic of T2DM. Consequently, the consumption of HFD for 8 weeks followed by a low dose STZ injection was developed to provide a more precise model of T2DM [124]. In this model, exposure to HFD leads to initial hyperinsulinemia to combat the diet-induced increase in blood sugar levels. STZ injection then results in partial removal of pancreatic β-cells, reducing overall insulin secretion. This reduction contributes to insulin resistance and, consequently, the development of DM [124]. This technique, used successfully with rodents, clearly shows more deleterious effects compared to HFD exposure alone [18,127], supporting use of the model and the synergistic effects of high blood sugar and high lipid levels characteristic of obese T2DM patients. 

HFD + STZ rats show reduced and delayed ERG b-waves 2 months after STZ injection, and vascular changes at 4 months [18]. 12 weeks (~3 months) after DM induction using HFD + STZ (Figure 4), the retinas exhibit thinning along with heightened oxidative stress, inflammatory markers, apoptotic cells, and an increase in acellular capillaries. Additionally, there is an observed loss of pericytes and vascular leakage [56]. These morphological and biochemical differences are still evident 37 weeks after STZ injection, when an increase in GFAP levels in Muller glia, a loss of retinal ganglion cells, an increase in RAGE expression in the inner nuclear and ganglion cell layers, and increases in both VEGF and TNFα levels [127] are found. 

Together, these T2DM rodent models show a DR progression where functional changes are evident early and before significant vascular pathology. This common progression reflects a specific time course for disease progression. Interestingly, the functional changes do not always correspond to morphological changes in the retina, though inflammation and oxidative stress clearly occur early in the disease and are likely to trigger subsequent complications.

### 2.2. Zebrafish Models

Zebrafish (*Danio rerio*) have emerged as a relatively recent addition to the study of DR and related DM complications compared to rodents. Much of the data collected from zebrafish related to hyperglycemia-related changes are also reported in rodents. However, zebrafish, unlike rodents, share a diurnal nature with humans and possess a similar retinal morphology characterized by the presence of both rod and cone photoreceptors to capture visual stimuli [71]. The similarities between zebrafish retinal morphology and physiology and that of both rodents and humans suggests that the zebrafish retina is a valuable tool for advancing our understanding of DR [20,71].

#### 2.2.1. Glucose Immersion

Glucose immersion is an efficient, simple, and quick method to induce hyperglycemia and DM symptoms [66] in zebrafish. This approach involves two related techniques: exposure to alternate immersion with stepwise increasing glucose concentrations for prolonged exposure [60,128], or chronic immersion with a constant glucose concentration for short term exposure [76,77,129]. These models have propelled diabetes research forward, enabling the maintenance of elevated blood glucose levels in adult zebrafish over extended periods, akin to human T2DM. 

**Figure 5:**
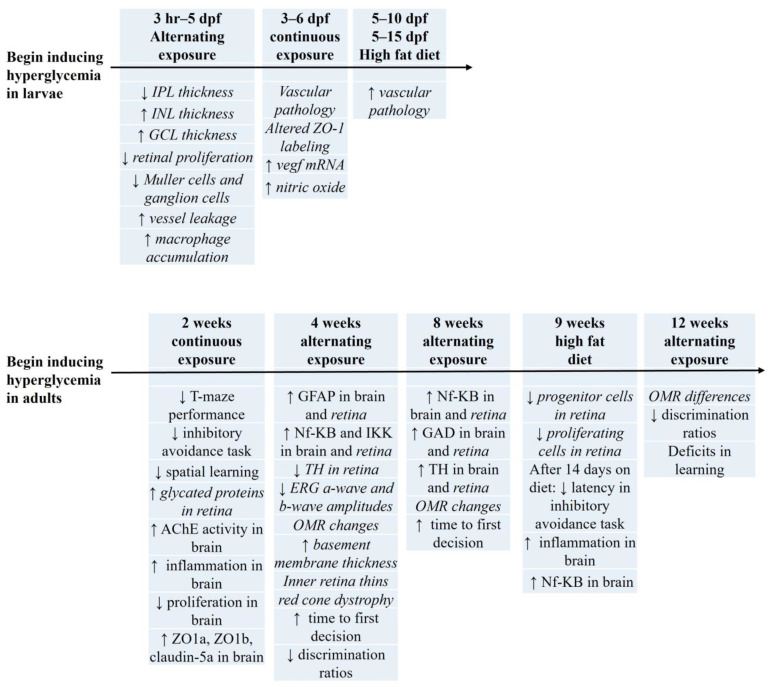
Hyperglycemia-induced changes in zebrafish models of T2DM. In zebrafish larvae and adults, hyperglycemia is induced using three mechanisms to replicate T2DM: alternate immersion in a glucose solution, continuous immersion in a glucose solution, or via a high fat diet (HFD). Using zebrafish larvae (top panel), both alternating and continued immersion in a glucose solution cause retinal complications. In contrast, exposure to a HFD for either 5 or 10 days affects ocular vessels (dpf = days postfertilization). Inducing hyperglycemia in adult zebrafish (bottom panel), retinal and brain changes are observed after 2 weeks of continuous exposure or after 4 weeks of alternating exposure. Adults fed a high fat diet, in contrast, show differences after 9 weeks on the diet. Each timeline begins with hyperglycemic induction (at the left). Outcomes, listed below the arrow/line, are given for each time point and for both panels the text in italics reflects changes in retina/vision, while the non-italic text reflects the changes in brain/cognition. ↑ = increase; ↓ = decrease. See text for details.

Work in our lab induces T2DM through alternate immersion in glucose [60]. Using this technique, we observe a significant reduction in the thickness of the inner plexiform and inner nuclear layers of retina after 1 month of hyperglycemia [60]. To induce hyperglycemia for a longer period, adult zebrafish are alternately exposed to a 1% glucose solution for 2 weeks, then to a 2% glucose solution for 2 weeks, and, finally to a 3% glucose solution for 4 weeks [78,128]. Blood sugar levels in glucose-treated fish average 3× higher than those in the control group, indicating successful induction of hyperglycemia after both 4 and 8 weeks of glucose exposure [128]. After 4 weeks of hyperglycemia, retinal tissue displays a decrease in tyrosine hydroxylase (TH) levels [83], but an increase in the levels of GFAP, NF-κB [81] and IKK [83], consistent with inflammation. ERG recordings show reductions in both a-wave and b-wave amplitudes [81]. Reductions in spectral ERGs are most pronounced for long wavelength cones [81], which correlates with the red cone dystrophy that occurs in 4 week hyperglycemic retinas [75]. Thickened basement membranes are also evident at this time point [75]. Extending hyperglycemia to 8 weeks [82,83] shows continued inflammation in retina as well as increases in both GAD (glutamic acid decarboxylase) and TH levels [83], suggesting neuronal deficits. Optomotor responses (OMRs) increased in glucose-treated fish after 4 and 8 weeks of exposure in one study [130], whereas another study notes significantly lower scores after 4, 8, and 12 weeks of exposure [82] (Figure 5).

Constant glucose immersion for 14 days (2 weeks) can also induce hyperglycemia [76,77] in adult zebrafish. Initial studies using this protocol monitored glycemic control in zebrafish by assessing non-enzymatic glycation of proteins [77]. This study reports that a continuous 14 day exposure to 2% glucose (111 mM) leads to a 40–41% increase in fructosamine levels that persist for 7 days following glucose withdrawal [77]. Zebrafish adults exposed to an alternating high (4%, 5%) glucose solution from 3 h to 5 days postfertilization (dpf) display an increase in sprouting of hyaloid blood vessels in the eye [20], suggesting persistent effects of developmental hyperglycemia. Examining these same larvae immediately after hyperglycemic induction (i.e., at 5 dpf) reveals significant changes in the thicknesses of the inner plexiform and the inner nuclear and ganglion cell layers of the retina, as well as an increase in macrophage accumulation, a decrease in proliferating cells, a decrease in the numbers of Muller glia and ganglion cells, and an increase in macrovascular complications [20]. Zebrafish larvae exposed to a higher glucose concentration (130 mM) from 3 to 6 dpf display thicker hyaloid-retinal vessels, changes in ZO-1 labeling, and increases in *vegf* and nitric oxide levels [131].

#### 2.2.2. Diet Induced Obesity/High Fat Diet

Zebrafish can become obese by consumption of a high fat diet and/or overfeeding for 4 to 8 weeks [69,132,133,134]. As in rodents, diet-induced obese (DIO) zebrafish display an increase in weight, BMI, cholesterol, and triglyceride levels compared to normal fed controls [133,135,136,137,138]. Adult DIO zebrafish are also hyperglycemic [134,135,136,138]. We were able to find only one study that addresses retinal changes in DIO hyperglycemic adult zebrafish. This study [137] reports that adult zebrafish overfed with *Artemia* nauplii (high-fat diet T2DM model) for 9 weeks have fewer progenitor and proliferating cells in the retina. Another study, using larval zebrafish fed a HFD + 3% glucose for 5 days starting at 5 dpf, shows changes to blood vessels in the eye that include increases in diameter and branching around the lens [139]. If zebrafish larvae are maintained on a HFD + 3% glucose for 10 days (until age 15 dpf), lipid accumulation, a reduction in blood flow, and a thicker endothelial layer within the optic artery are observed [140]. 

Overall, T2DM zebrafish models display similar characteristics to rodents. Inflammation, functional deficits, and vascular changes are all evident. Glucose-induced changes in zebrafish retina occur after 2 weeks (continued exposure) or 4 weeks (alternating exposure) of treatment. Adults that are alternately exposed to glucose for 4 weeks show functional and vascular changes, similar to the morphological and vascular changes noted in larvae. These results suggest a different onset of complications in zebrafish compared to rodent retinas.

## 3. Hyperglycemia-Induced Changes in Brain

### 3.1. Rodent Models

Many recent studies have established a strong correlation between T2DM and cognitive decline. This connection has been studied in depth using *db*/*db* mice, GK and ZDF rats, and HFD-induced hyperglycemia. Across these models, the hippocampus has been suggested as a vulnerable brain region in T2DM [141,142,143].

Genetic models of T2DM show consistent degradation of cognition. In *db*/*db* mice (Figure 1), performance decreases in the Morris Water Maze (8, 10, and 14 week old animals) [144,145,146] and the Y-Maze (10 week old) [144] tasks. GK rats (Figure 2) show decreased learning and memory consolidation on the Barnes Maze at 4 months of age [143] and a decrease in spatial cognition and exploratory behavior in the Y-maze task at 7 weeks of age [147]. ZDF rats (Figure 3) show decreases in latencies in the passive avoidance task at 8 weeks of age [148] and these deficits continue with longer delays in a go no/go task at 4 months of age [149]. These results suggest that in rodent genetic models of T2DM, there are consistent behavioral outcomes, which may suggest a common cellular mechanism.

In contrast, the literature is conflicting with regard to HFD-associated cognitive deficits. For example, mice maintained on a HFD (composed of 60% fat) for 17 days have a reduction in the number of dendrites and spines in the hippocampal CA3 region, which correlates with a decline in spatial working memory in a T-maze task [141] (Figure 4). HFD-fed mice (60% fat for 14 weeks) also show decreases in spatial learning and memory modeled using the Barnes Maze task [150]. A longer duration on the HFD (54% fat for 8 months) leads to a decrease in performance on odor detection tasks [151]. In contrast, the HFD had no impact on memory consolidation that is assessed using tasks such as the Morris Water Maze (HFD: 60% fat for 6.5 months) [152] or the passive avoidance and water T-maze (HFD: 60% fat for 9 weeks) [153]. Given that the fat content of the diet is similar across these studies, the different outcomes could be due to differences in the duration of exposure and/or the behavioral test used. Alternatively, there could be a mechanistic contribution that may lead to the variable reactions. 

Altered permeability of the BBB could be one of the driving forces behind the impaired cognition observed in DM rodent models. However, much like the HFD findings, there is some controversy about whether hyperglycemia increases BBB permeability. Some studies report neither increases in BBB permeability [154,155] nor morphological changes in brain vasculature [155]. Other researchers, however, report an increase in BBB permeability in relation to specific cognitive deficits [144,148,156,157], which could be due to secondary inflammation and oxidative stress [148]. Hyperglycemic rodents with higher BBB permeability also tend to have an increase in neuroinflammation [144,156,157]. Further, memory loss is directly correlated to both blood glucose levels and BBB permeability [144]. These models provide support for parallel changes in retina (BRB) and brain (BBB) and are a good base to study the biological mechanisms surrounding the mechanisms between T2DM and cognitive decline.

Despite their clear role, neuroinflammation and BBB permeability are not the only explanations for cognitive decline. There are reports identifying a lowered cholinergic system in the hippocampus of ZDF rats [158], *db*/*db* mice, and postmortem human brains [146], which is associated with increased apoptosis. A decrease in the Akt pathway leading to a decrease in cell growth and proliferation [141] is also associated with hyperglycemia. HFD and ZDF rats also show an increase in anxiety and dysregulated mood processes, suggesting a role for lipidemia in higher order functions in addition to cognition [148,150].

### 3.2. Zebrafish Models

Zebrafish do not have a defined hippocampus or the trisynaptic circuitry of mammals that is crucial for spatial learning and memory. However, zebrafish do have analogous brain structures that play a functionally similar role allowing them to have complex cognition [159,160]. The lateral pallium, for example, might serve a similar role in spatial learning to the mammalian hippocampus, and the medial pallium may be equivalent to the mammalian amygdala [159,160]. Zebrafish can also gain and express spatial learning, which is attributed to the hippocampus [159]. Habituation learning, conditioned place preference, passive and active avoidance, spatial alterations, spatial and visual discrimination tasks, and stimulus generalization tasks have all been modified and are used with zebrafish. The zebrafish model is also being used to show that hyperglycemia impairs cognitive function [161]. 

While there are some genetic models of diabetes in zebrafish, they are mostly Type 1 DM models. Consequently, hyperglycemia associated with T2DM is primarily induced in the fish through bath application [66] of glucose. Inducing hyperglycemia via continuous exposure of 111 mM (2%) glucose for 14 days increases acetylcholinesterase activity associated with memory deficit [76,77] and causes cognitive dysfunction in T-maze [162] and inhibitory avoidance tasks [76] (Figure 5). Similarly, continuous immersion in 83.25 mM sucrose for 14 days decreases cognitive function in both the T-maze and the spatial learning tasks [163]. Fish exposed to a HFD (26% fat for 14 days) also show memory decline based on a decrease in latencies in an inhibitory avoidance task [164]. Using the three-chamber choice discrimination task and a stepwise alternating glucose immersion system (1% for 2 week, 2% for 2 weeks, 3% for 4 week, and 4% for 4 weeks), we observe impaired cognition that lasts beyond 14 days and up to 12 weeks [82,83]. 

As suggested in rodents, cognitive deficits in T2DM zebrafish models are hypothesized to be due to an increase BBB permeability and neuroinflammation. Hyperglycemic zebrafish continuously exposed to 111 mM glucose for 14 days show increases in the tight junction proteins ZO-1 and claudin-5 in brain [22], which would alter BBB permeability. However, and like rodents, other studies report no correlation between BBB permeability and hyperglycemia [135] in zebrafish, though there is an increase in oxidative stress [135]. Increased oxidative stress is correlated with BBB permeability and neuroinflammation in T2DM zebrafish models [136,162], suggesting a possible mechanism. DIO zebrafish, as well as those either continuously or alternately exposed to glucose, show an upregulation of the proinflammatory cytokines, *il1β*, *il6*, *il8*, and *tnfα* in brain tissues [22,136]. NF-κB levels are also increased in DIO [136] and alternately exposed fish [83]. Further, changes to the cholinergic system are implicated as a mechanism underlying decreases in cognition in zebrafish [76]. Hyperglycemic zebrafish also display increases in anxiety and aggression [76,164], with less motivation and increases in depression [162]. This is very similar to what humans with dementia and Alzheimer’s undergo later in life, as well as some T2DM rodent models.

Together, rodent and zebrafish models suggest possible mechanistic causes behind the cognitive deficits that are correlated with T2DM. Further, the behavioral deficits and cellular mechanisms in zebrafish mirror those found in rodent studies. These same mechanisms can be seen in the breakdown pathways of DR, suggesting global damage caused by neuroinflammation due to sustained blood glucose levels.

## 4. Correlation of Hyperglycemia-Related Changes in Retina and Brain

**Figure 6:**
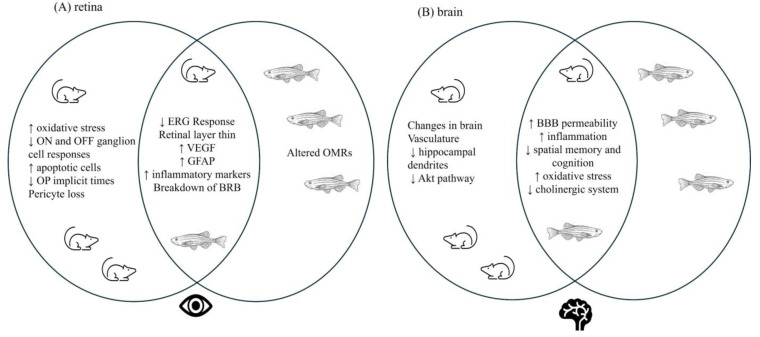
Summary figure comparing hyperglycemia-associated changes in retina and brain. Hyperglycemia-induced changes are observed in both the retina and brain in zebrafish and rodent models of T2DM. Venn Diagrams show similarities and differences across the different models. For the retina (**A**) and brain (**B**), some reported changes occur only in rodent models, while others are observed in both rodents and zebrafish. Interestingly, the only hyperglycemia-induced vision related outcome that is specific to zebrafish is a change in optomotor responses (OMRs). Importantly, there are several shared mechanisms in brain and retina across species, suggesting conserved pathological mechanism(s) in these T2DM models. ↑ = increase; ↓ = decrease.

Prolonged hyperglycemia, characteristic of individuals with T2DM, deleteriously affects both retina and brain, leading to vision and cognitive deficits. Common pathologies observed in both tissues across rodent and zebrafish models (Figure 6) include increased inflammation and vascular changes, which are hypothesized to then impact neuronal function. However, in rodent (i.e., *db*/*db* mouse, GK rat, and ZDF rat) retinas, neuroinflammation and oxidative stress occur first, before vascular differences. Determining the precise timing of these outcomes in the brain has proved more challenging. Functional deficits in retina are typically measured physiologically, while cognitive function is determined behaviorally. This latter difference could make the identification of disease progression difficult as behavioral effects may take longer to appear. This concern could be eliminated by using the same animal model to study both hyperglycemia-associated retinopathy and cognitive decline, as it would determine if changes occur in parallel or if changes in one tissue precede changes in the other. Indeed, the consistently observed differences in vascular pathology and neuroinflammation between the retina and the brain, together with functional differences, suggest that hyperglycemia-induced retinal changes, which are more easily observed, could serve as an indicator of corresponding changes in the brain [13,165].

However, caution is advised. To date, there are few investigations that have examined both visual and cognitive dysfunction in the same T2DM study using either zebrafish or rodent models. In zebrafish, continuous exposure to 2% glucose for 14 days increases protein glycation in retina, increases brain acetylcholinesterase activity, and reduces memory performance in an inhibitory avoidance task [76,77]. These results suggest that the continued hyperglycemia is concurrently affecting both the brain and the retina. Similar results have been obtained from glucose immersion experiments. Adult zebrafish exposed to alternating hyperglycemia for 4, 8, and 12 weeks display significant differences in OMR scores at all timepoints [82,83]. After 4 weeks of hyperglycemia, there are also reductions in photopic ERG responses [81], decreases in cognition [82,83], and increases in inflammatory markers (Rel-A/NF-κB, IKK, GFAP) in both the retina and the brain. After 8 weeks of hyperglycemia, Rel-A/NF-κB protein levels are still elevated in the zebrafish retina and brain, as are GAD and TH levels, suggesting neuronal differences [83]; decreases in memory are observed after 8 and 12 weeks [82,83]. In contrast, a recent study [95] examining retinal and cognitive outcomes in GK rats from 1–8 months of age reports functional deficits in the retina at 1 month, whereas cognitive deficits are not evident until 6 months of age. In this rodent model, therefore, hyperglycemia-associated retinal changes occur prior to cognitive differences [95].

For the other rodent T2DM models, determining a sequence of hyperglycemia-associated changes in retina and brain is more difficult. While different research labs may use the same T2DM rodent model, the age, outcome(s), and techniques used are not always the same, making comparisons problematic. However, to date, such a comparison is the best way to determine if hyperglycemia-induced retinal changes precede or follow brain changes. In examining Figure 1, Figure 2, Figure 3 and Figure 4, we find that the time course of hyperglycemia-associated changes in retina and brain varies depending on the T2DM model used. In *db*/*db* mice, functional and morphological changes in retinal tissue are observed at 8–10 weeks of age, when there is also a decrease in performance in the Morris water maze and Y-maze tasks. These results suggest that hyperglycemia-associated visual and cognitive deficits occur concurrently in *db*/*db* mice, as they do in zebrafish. In contrast, in GK rats, differences in ERGs are apparent at 1 month (4 weeks) of age [95,107] while cognitive deficits are observed later, beginning at 7 weeks [107] and still evident at 6 months [95], which supports the above longitudinal study showing that retinal changes precede cognitive deficits [95]. The retinas of ZDF rats display oxidative stress and mitochondrial changes when prediabetic (5–7 weeks), with changes in cognitive behaviors first reported at 8 weeks of age, again suggesting that retinal pathology occurs first. However, rats fed a HFD show early cognitive deficits (after 17 days on the diet), with retinal changes occurring later, after 8 weeks on the diet. 

Despite the differences in timing across the different animal models, the specific cellular mechanisms triggered by hyperglycemia are strongly conserved. Inflammation, glial responses, and oxidative stress are consistently reported. ERG differences occur early, typically preceding vascular changes, in some models by several weeks. Genetic models of T2DM lead to early hyperglycemia, while non-genetic/induced models develop hyperglycemia more slowly. In younger animals, retinal deficits may be evident without any changes to the brain; meanwhile, in older animals, cognitive deficits are more frequently reported, suggesting an age-dependent component. 

There are also correlations reported between T2DM, cognitive decline, and DR in human patients. Cognitive decline in humans is associated with whole brain atrophy [166,167], lower grey and white matter volumes, and an increase in white matter lesions [167,168,169,170]. Atrophy in the hippocampus, amygdala, and cerebellum [166,168,170,171] is also reported. Behaviorally, T2DM patients show decreases in processing speed [166,168,169,170,171,172], memory [168,169,170,171,172,173], attention [168,170,171], executive function [166,168,169,170,171,172], and mental flexibility and speed [168,173]. In these individuals, microvascular complications such as DR are considered good markers of the onset and severity [167,168,172,173,174] of cognitive deficits. 

As many as 21–39% of T2DM patients have some form of DR [175], and these rates can increase to up to 60% 20 years after first diagnosis [176]. Clinically, DR is diagnosed by vascular changes. The initial stage is non-proliferative diabetic retinopathy (NPDR), which is characterized by increases in vascular permeability, capillary occlusions, and microaneurysms [65,153,175,177,178,179]. Most animal models mimic this stage. As DR progresses, it becomes proliferative (PDR) as blood vessels grow into the retina [65,153] and blindness occurs. In the early stages of DR, thickness of the nerve fiber layer [65], cornea nerve density, and corneal sensitivity decrease [180] and the basement membrane thickens [181]. DR has both vascular and neural components that most likely become relevant before NPDR is diagnosed, which is why animal models are important for the study of the early stages of this disease [176].

While rodent and zebrafish T2DM models clearly show shared hyperglycemia-induced pathologies that are translationally relevant, there is clearly a need for additional work that assesses, in parallel, the visual and cognitive outcomes of hyperglycemia across different ages. These longitudinal studies, performed in the same animal model, will provide a clearer picture about the impact of long term hyperglycemia in both tissues. 

## 5. Conclusions

We have complied a comprehensive overview of the existing literature concerning visual and cognitive impairments observed in rodents and zebrafish models of T2DM. Our analysis reveals parallel pathologies in both the retina and the brain across these animal models. Further we found that hyperglycemia-induced retinopathy follows a clear (and consistently reported) progression that involves early inflammation and/or oxidative stress that is followed by functional impairment and vascular and morphological pathologies. The disease progression in the brain is not as well established. This is due, in part, to the larger number of studies that focus on DR, and to the ease of access to the retina. However, inflammation and vascular impairment are still observed in brain tissue. Studies looking at both visual and cognitive markers concurrently, at multiple timepoints, can give us more information about how one macrovascular complication is impacting the other. Overall, it is clear that both zebrafish and rodents have good predictive validity for visual and cognitive decline. Diabetic retinopathy and cognitive decline have very similar pathologies; therefore, looking further into the predictive nature of the decline is the clear next step going forward. 

## Data Availability

Not applicable.
